# Spontaneous Pregnancy After Laparoscopic Treatment of Ovarian Endometrioma: A Retrospective Cohort Study Comparing Stripping and Ethanol Sclerotherapy

**DOI:** 10.7759/cureus.117643

**Published:** 2026-10-08

**Authors:** Guy Lambert M Monzango Sibo, Gracia K Ntanga

**Affiliations:** 1 Gynecology, University of Kinshasa, Kinshasa, COD; 2 Gynecology, Cliniques Universitaires de Kinshasa, Kinshasa, COD

**Keywords:** endometriosis fertility index, ethanol sclerotherapy, laparoscopy, ovarian endometrioma, ovarian reserve, spontaneous pregnancy, stripping

## Abstract

Ovarian endometrioma is associated with infertility, whereas its treatment may compromise ovarian reserve. This retrospective cohort evaluated factors associated with spontaneous pregnancy after laparoscopic treatment of ovarian endometrioma, with particular attention to the Endometriosis Fertility Index (EFI) and reproductive outcomes after cystectomy using the stripping technique vs ethanol sclerotherapy. Forty-eight infertile women with histologically confirmed ovarian endometrioma and revised American Society for Reproductive Medicine stage III-IV endometriosis underwent laparoscopic treatment between 2022 and 2024 in Kinshasa, Democratic Republic of the Congo, with follow-up through June 2026; 33 underwent cystectomy, and 15 underwent ethanol sclerotherapy. Overall, 29/48 women (60.4%) had primary infertility, 30/48 (62.5%) had bilateral endometriomas, and 29/48 (60.4%) had stage IV disease. Spontaneous pregnancy occurred in 17/48 women (35.4%), and 11/48 (22.9%) achieved a live birth. Pregnancy occurred in 9/15 women (60.0%) after sclerotherapy vs 8/33 (24.2%) after cystectomy (p = 0.024). Among women with EFI ≥7, 15/26 (57.7%) conceived, compared with 2/22 (9.1%) with EFI <7 (p < 0.001). In the adjusted Cox model, EFI ≥7 (adjusted hazard ratio (aHR) 7.96, 95% confidence interval (CI) 1.78-35.57; p = 0.007) and ethanol sclerotherapy (aHR 3.11, 95% CI 1.17-8.29; p = 0.023) were associated with a higher rate of spontaneous pregnancy. Both ovarian-reserve markers improved in 15/15 women (100.0%) after sclerotherapy vs 9/33 (27.3%) after cystectomy (p < 0.001). These findings suggest that a favorable EFI identifies women with a higher likelihood of spontaneous pregnancy and that ethanol sclerotherapy may offer reproductive advantages over cystectomy; however, the retrospective design, small sample, and nonrandomized treatment allocation limit causal inference.

## Introduction

Endometriosis is a chronic inflammatory disease affecting approximately 10% of women of reproductive age and is particularly prevalent among women undergoing evaluation for infertility [1]. Ovarian endometriomas represent a major phenotype of endometriosis and may impair fertility through inflammation, oxidative stress, fibrosis, altered folliculogenesis, distorted tubo-ovarian anatomy, and loss of functional ovarian tissue [1,2]. In women with infertility, the therapeutic dilemma is therefore twofold: the disease itself can compromise reproductive potential, whereas treatment, particularly excisional ovarian surgery, may further reduce ovarian reserve.

The European Society of Human Reproduction and Embryology recommends cystectomy rather than simple drainage and coagulation when surgery for an endometrioma is indicated because of better control of recurrence and pain; however, the guideline emphasizes minimizing ovarian damage and incorporating age, previous surgery, ovarian reserve, patient preferences, and other fertility-related factors into treatment decisions [2]. The Endometriosis Fertility Index (EFI), developed by Adamson and Pasta, is a validated tool for predicting pregnancy without in vitro fertilization after endometriosis surgery [3].

The stripping technique remains widely used because it removes the cyst wall and provides tissue for histopathologic assessment. Nevertheless, several studies and meta-analyses have demonstrated postoperative reductions in anti-Müllerian hormone (AMH) and other markers of ovarian reserve after cystectomy [4-9]. Ovarian injury may result from inadvertent removal of the normal ovarian cortex, thermal hemostasis, or disruption of ovarian vascularization. Less destructive approaches, including laser ablation and sclerotherapy, have therefore attracted increasing interest in women for whom preservation of ovarian reserve is a priority [10-14].

Sclerotherapy treats the inner cyst lining without extensive stripping of the ovarian cortex. Systematic reviews suggest that it may better preserve postoperative AMH and oocyte yield than cystectomy, although evidence regarding spontaneous pregnancy and live birth remains heterogeneous and subject to selection bias [11-16]. At the same time, the probability of spontaneous conception after surgery depends on individual and operative factors that the EFI is designed to integrate [3]. Data from sub-Saharan Africa remain scarce.

The primary objective of this study was to determine the rate and timing of spontaneous pregnancy after laparoscopic treatment of ovarian endometrioma and to identify factors associated with conception, particularly the EFI. The secondary objective was to compare reproductive outcomes and changes in ovarian reserve between women treated with stripping and those treated with ethanol sclerotherapy.

## Materials and methods

Study design and setting

We conducted a retrospective cohort study using clinical and follow-up data from women treated for ovarian endometrioma at Onyx Medical Center and the Clinique Présidentielle de l’Union Africaine (CPUA), Kinshasa, Democratic Republic of the Congo. The surgical team performed the procedures between January 2022 and December 2024, and reproductive follow-up continued through June 2026. Data were extracted from medical records, operative reports, and available follow-up documentation. The manuscript was prepared in accordance with the Strengthening the Reporting of Observational Studies in Epidemiology (STROBE) recommendations [17]. The final analytic cohort included 48 women with the variables required for time-to-pregnancy analysis and EFI calculation.

Study population

Eligible participants were women of reproductive age who had infertility associated with an ovarian endometrioma and underwent laparoscopic treatment, revised American Society for Reproductive Medicine (rASRM) stage III or IV endometriosis, histopathologic confirmation of ovarian endometriosis, a desire for pregnancy after treatment, and documented follow-up for the primary outcome. The clinical protocol excluded couples with a major non-endometriosis infertility factor that precluded a reasonable attempt at natural conception, including severe male-factor infertility. The analyzed cohort comprised 48 women.

Preoperative assessment

Recorded variables included age, body mass index, gravidity, parity, primary or secondary infertility, duration of infertility, previous ovarian cystectomy, previous appendectomy, unilateral or bilateral endometrioma, and rASRM stage. Transvaginal ultrasonography documented three orthogonal dimensions of each endometrioma; cyst volume was estimated using the ellipsoid formula (length × width × anteroposterior diameter × 0.523), and volumes were summed in bilateral disease. Ovarian reserve was assessed using serum AMH (ng/mL) and antral follicle count (AFC) before treatment and again at six months.

Calculation of the EFI

The EFI was calculated on a 0-10 scale according to the system described by Adamson and Pasta [3]. It combines historical factors, including age, duration of infertility, and previous pregnancy, with surgical factors, including the least function score (which evaluates residual function of the fallopian tubes, fimbriae, and ovaries at the end of surgery), the rASRM endometriosis lesion score, and the total rASRM score. Surgical EFI components were derived from operative findings documented in the study database. Higher scores indicate a more favorable prognosis for pregnancy without assisted reproductive technology. For the present analysis, EFI ≥7 was considered a favorable prognostic score. No table, figure, scoring template, or other copyrighted material from the original EFI publication was reproduced or adapted in this manuscript; the published method is cited solely to describe calculation of the score from the study data.

Laparoscopic treatment

All procedures were performed by the same surgical team using a fertility-sparing laparoscopic approach. In the stripping group, the endometrioma was opened, and the cyst wall was separated from the ovarian cortex using traction-countertraction, with conservative hemostasis intended to minimize thermal injury.

In the ethanol sclerotherapy group, after laparoscopic identification and exposure of the ovarian endometrioma, the cyst was punctured, and its contents were completely aspirated, with the evacuated volume measured. A cyst-wall biopsy was obtained before instillation of the sclerosant. A Foley catheter was then introduced through an abdominal trocar into the cyst cavity, and the balloon was inflated to secure the catheter intracystically and maintain a seal during instillation. Sterile non-denatured 99% ethanol, corresponding to two-thirds of the volume initially aspirated from the endometrioma, was slowly instilled through the Foley catheter under direct laparoscopic visualization. After a 15-minute contact period, the ethanol was completely re-aspirated, the balloon was deflated, and the catheter was removed. The ovary and pelvic cavity were subsequently inspected to confirm hemostasis and identify any evidence of ethanol leakage. Cyst-wall biopsy specimens were submitted for histopathologic examination; ovarian endometriosis was confirmed in all included women.

Outcomes and follow-up

The primary outcome was spontaneous pregnancy during follow-up, defined as a pregnancy achieved without assisted reproductive technology after laparoscopic treatment. Time to pregnancy was calculated in days from the procedure to the documented conception. Women who did not conceive were censored at the date of their last recorded reproductive follow-up. Secondary outcomes included live birth, miscarriage, ongoing pregnancy at the end of follow-up, and changes in ovarian-reserve markers at six months. Combined improvement in ovarian reserve required a postoperative increase in both AMH and AFC relative to baseline.

Statistical analysis

Continuous variables were summarized as mean ± standard deviation when appropriate or as median and interquartile range (IQR). Categorical variables were reported as counts and percentages. Between-group comparisons used the Mann-Whitney U test for continuous variables and Fisher’s exact test for categorical variables. Paired pre- and postoperative ovarian-reserve measurements were compared using the Wilcoxon signed-rank test.

Time to spontaneous pregnancy was analyzed using Kaplan-Meier methods, with women who did not conceive treated as censored observations. Curves were compared using the log-rank test. Cox proportional hazards models were used to estimate hazard ratios (HRs) and 95% confidence intervals (CIs). Given the limited number of pregnancy events (n = 17), the multivariable models were intentionally parsimonious. The primary model included favorable EFI (≥7) and treatment technique. A sensitivity model included age, preoperative AFC, and treatment technique but excluded EFI to avoid overadjustment, as age and reproductive history contribute to EFI. An additional analysis modeled EFI as a continuous variable. The proportional hazards assumption was explored using Schoenfeld residuals. Statistical significance was defined as a two-sided p < 0.05. All analyses were performed using Python version 3.13.5 (Python Software Foundation, DE, USA), with statsmodels version 0.14.6 (Statsmodels Developers, USA), SciPy version 1.17.0 (SciPy Developers, USA), and NumPy version 2.3.5 (NumPy Developers, USA).

Ethical considerations

The Ethics Committee of the School of Public Health, University of Kinshasa, approved the study (approval number: ESP/CE/687/2026), and it was conducted in accordance with the Declaration of Helsinki.

## Results

Participant characteristics

The database included 48 women. The mean age was 30.0 ± 4.1 years, and the median body mass index was 25.1 kg/m² (IQR 23.2-27.7). Twenty-nine women (60.4%) had primary infertility and 19 (39.6%) had secondary infertility. Median infertility duration was 3.9 years (IQR 2.6-5.3). Nineteen women (39.6%) had undergone previous ovarian cystectomy, and 15 (31.3%) had a history of appendectomy. Endometriomas were bilateral in 30 women (62.5%), and 29 women (60.4%) had rASRM stage IV disease. Median total endometrioma volume was 88.7 mL (IQR 54.4-120.1). Median EFI was 7 (IQR 6-7), and 26 women (54.2%) had EFI ≥7.

Thirty-three of 48 women (68.8%) underwent stripping, and 15/48 (31.3%) underwent ethanol sclerotherapy. Women in the sclerotherapy group were younger (median 27.0 vs 32.0 years; p = 0.028), had a higher preoperative AFC (13 vs 10; p = 0.017), and had a higher EFI (median 7 vs 6; p = 0.012). An EFI ≥7 was present in 12/15 women (80.0%) in the sclerotherapy group compared with 14/33 (42.4%) in the stripping group (p = 0.027). Other measured baseline characteristics did not differ significantly between groups (Table 1).

**Table 1:** Baseline characteristics according to treatment technique p-values compare stripping with ethanol sclerotherapy. * Statistically significant (p < 0.05). IQR: interquartile range, rASRM: revised American Society for Reproductive Medicine, AMH: anti-Müllerian hormone, AFC: antral follicle count, EFI: Endometriosis Fertility Index

Characteristic	Overall (n = 48)	Stripping (n = 33)	Ethanol sclerotherapy (n = 15)	p-value
Age, years, median (IQR)	30.5 (26.0-33.0)	32.0 (26.0-34.0)	27.0 (25.0-30.5)	0.028*
Body mass index, kg/m², median (IQR)	25.1 (23.2-27.7)	25.3 (23.8-27.8)	23.7 (23.0-26.9)	0.410
Primary infertility, n (%)	29 (60.4)	21 (63.6)	8 (53.3)	0.538
Infertility duration, years, median (IQR)	3.9 (2.6-5.3)	4.4 (2.6-5.8)	3.5 (2.5-4.7)	0.256
Previous ovarian cystectomy, n (%)	19 (39.6)	11 (33.3)	8 (53.3)	0.218
Previous appendectomy, n (%)	15 (31.3)	12 (36.4)	3 (20.0)	0.328
Bilateral endometrioma, n (%)	30 (62.5)	23 (69.7)	7 (46.7)	0.198
rASRM stage IV, n (%)	29 (60.4)	21 (63.6)	8 (53.3)	0.538
Preoperative AMH, ng/mL, median (IQR)	2.12 (1.67-2.46)	2.01 (1.65-2.41)	2.25 (1.91-2.64)	0.161
Preoperative AFC, median (IQR)	11 (9-13)	10 (8-12)	13 (11.5-14)	0.017*
Total endometrioma volume, mL, median (IQR)	88.7 (54.4-120.1)	91.5 (57.3-136.5)	76.9 (50.6-101.6)	0.148
EFI, median (IQR)	7 (6-7)	6 (6-7)	7 (7-7)	0.012*
EFI ≥7, n (%)	26 (54.2)	14 (42.4)	12 (80.0)	0.027*

Ovarian-reserve outcomes

Across the entire cohort, median AMH increased from 2.12 ng/mL (IQR 1.67-2.46) before treatment to 2.38 ng/mL (IQR 1.78-2.85) at six months (Wilcoxon p < 0.001), while median AFC increased from 11 (IQR 9-13) to 12 (IQR 10-15; p < 0.001). These changes were driven predominantly by the sclerotherapy group.

After stripping, median AMH changed from 2.01 to 1.91 ng/mL (p = 0.376), whereas AFC increased from 10 to 11 (p = 0.003). After ethanol sclerotherapy, median AMH increased from 2.25 to 3.01 ng/mL (p < 0.001), and AFC increased from 13 to 15 (p < 0.001). The mean change in AMH was +0.75 ng/mL after sclerotherapy vs +0.03 ng/mL after stripping (between-group p < 0.001), and the mean change in AFC was +3.13 vs +0.52 follicles (p < 0.001). Combined improvement in ovarian-reserve markers was observed in all 15 women treated with sclerotherapy (100%) compared with nine of 33 women treated with stripping (27.3%; p < 0.001) (Table 2).

**Table 2:** Six-month ovarian-reserve outcomes Between-group p-value, where applicable. Within-group paired p-values: stripping, AMH p = 0.376 and AFC p = 0.003*; ethanol sclerotherapy, AMH p < 0.001* and AFC p < 0.001*. * Statistically significant (p < 0.05). AFC: antral follicle count, AMH: anti-Müllerian hormone, IQR: interquartile range

Parameter	Overall	Stripping	Ethanol sclerotherapy	p-value
Preoperative AMH, ng/mL, median (IQR)	2.12 (1.67-2.46)	2.01 (1.65-2.41)	2.25 (1.91-2.64)	0.161
AMH at 6 months postoperatively, ng/mL, median (IQR)	2.38 (1.78-2.85)	1.91 (1.71-2.44)	3.01 (2.70-3.44)	-
Mean change in AMH, ng/mL	+0.25	+0.03	+0.75	<0.001*
Preoperative AFC, median (IQR)	11 (9-13)	10 (8-12)	13 (11.5-14)	0.017*
AFC at 6 months postoperatively, median (IQR)	12 (10-15)	11 (10-13)	15 (14-17)	-
Mean change in AFC	+1.33	+0.52	+3.13	<0.001*
Combined ovarian-reserve improvement, n (%)	24 (50.0)	9 (27.3)	15 (100)	<0.001*

Spontaneous pregnancy and pregnancy outcomes

Seventeen of 48 women conceived spontaneously during follow-up, corresponding to a crude spontaneous pregnancy rate of 17/48 (35.4%). Among women who conceived, the median time to pregnancy was 187 days (IQR 148-238; range 82-336), approximately six months. Eleven of the 48 women achieved a live birth (22.9% of the entire cohort), corresponding to 11/17 pregnancies (64.7%). Three women experienced miscarriage, and three had an ongoing pregnancy at the end of follow-up.

Spontaneous pregnancy occurred in 9/15 women after ethanol sclerotherapy (60.0%) compared with 8/33 women after stripping (24.2%; p = 0.024). Among women who conceived, the median time to pregnancy was shorter after sclerotherapy (148 days, IQR 121-169) than after stripping (246.5 days, IQR 217.5-280.8; p < 0.001). Live birth occurred in 6/15 women after sclerotherapy (40.0%) and 5/33 after stripping (15.2%; p = 0.074) (Table 3).

**Table 3:** Reproductive outcomes after laparoscopic treatment Percentages are calculated using the corresponding column denominator (overall cohort, stripping group, or ethanol sclerotherapy group). * Statistically significant (p < 0.05). IQR: interquartile range

Reproductive outcome	Overall (n = 48)	Stripping (n = 33)	Ethanol sclerotherapy (n = 15)	p-value
Spontaneous pregnancy, n (%)	17 (35.4)	8 (24.2)	9 (60.0)	0.024*
Time to pregnancy, days, median (IQR)	187 (148-238)	246.5 (217.5-280.8)	148 (121-169)	<0.001*
Live birth, n (%)	11 (22.9)	5 (15.2)	6 (40.0)	0.074
Miscarriage, n (%)	3 (6.3)	2 (6.1)	1 (6.7)	-
Ongoing pregnancy at final follow-up, n (%)	3 (6.3)	1 (3.0)	2 (13.3)	-

The Kaplan-Meier cumulative probability of spontaneous pregnancy was 8/48 (16.7%) by six months and 17/48 (35.4%) by 12 months. No additional spontaneous pregnancies were recorded after day 336, so the curve plateaued thereafter. The overall curve and treatment-specific curves are shown in Figure 1A-1B.

**Figure 1:**
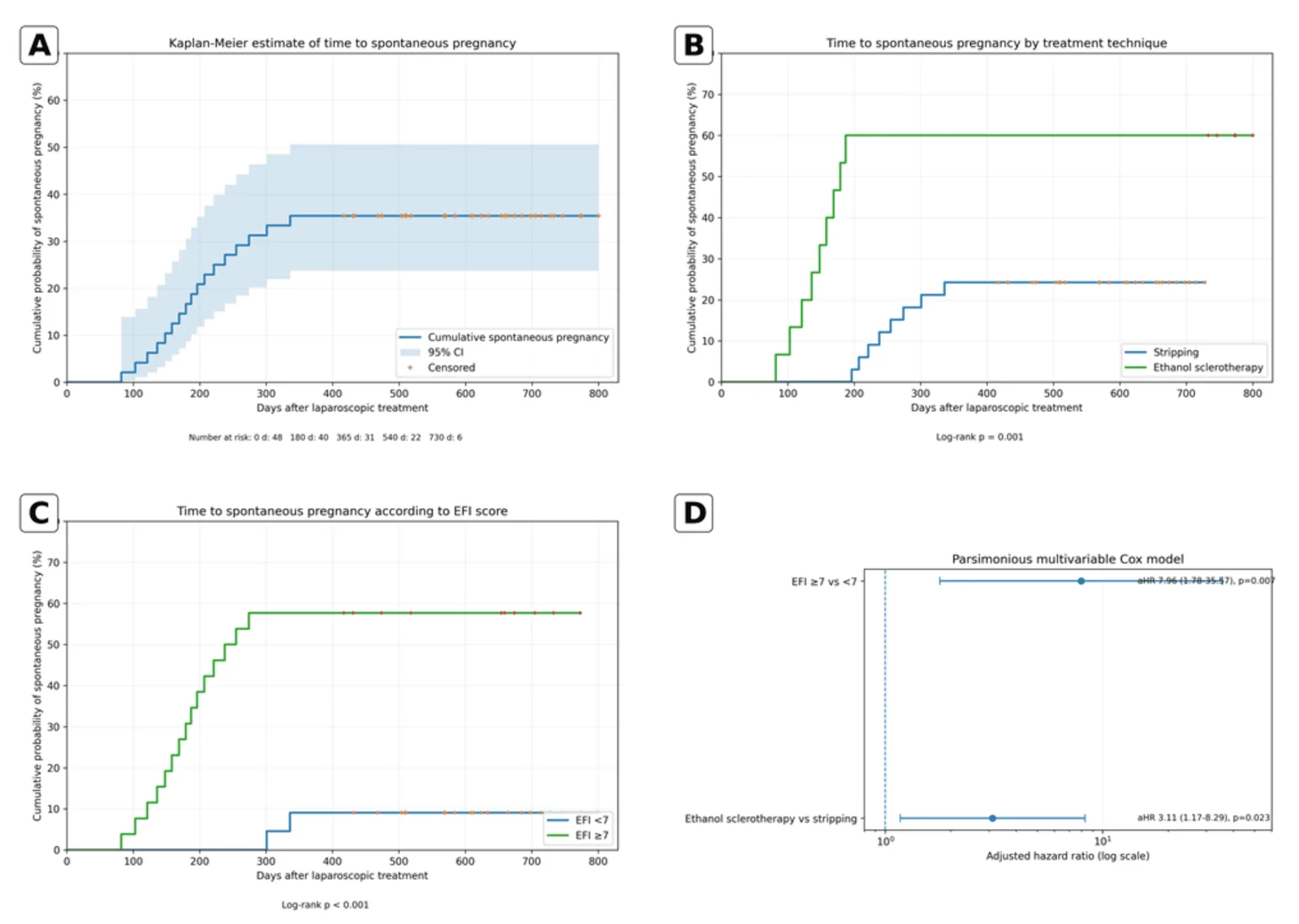
Time-to-pregnancy analyses and adjusted model (A) Kaplan-Meier estimate of the cumulative probability of spontaneous pregnancy in the entire cohort; 8/48 women (16.7%) had conceived by six months and 17/48 (35.4%) by 12 months. (B) Kaplan-Meier curves by treatment technique; 9/15 women (60.0%) conceived after ethanol sclerotherapy vs 8/33 (24.2%) after stripping (log-rank p = 0.001). (C) Kaplan-Meier curves by EFI category; 15/26 women (57.7%) conceived with EFI ≥7 vs 2/22 (9.1%) with EFI <7 (log-rank p < 0.001). (D) Forest plot of the primary Cox model showing adjusted associations of EFI ≥7 and ethanol sclerotherapy with time to spontaneous pregnancy. EFI: Endometriosis Fertility Index, HR: hazard ratio, CI: confidence interval

EFI and spontaneous pregnancy

The EFI was significantly higher in women who conceived than in those who did not (median 7 (IQR 7-8) vs 6 (IQR 6-7); p < 0.001). Among 26 women with EFI ≥7, 15/26 (57.7%) achieved spontaneous pregnancy, compared with 2/22 (9.1%) among women with EFI <7 (p < 0.001). The 12-month cumulative probability reflected these observed event proportions, with significant separation of Kaplan-Meier curves (log-rank p < 0.001) (Table 4; Figure 1C).

**Table 4:** Reproductive outcomes according to EFI score Time-to-pregnancy comparisons are descriptive in the EFI <7 group because only two pregnancies occurred. * Statistically significant (p < 0.05). EFI: Endometriosis Fertility Index, IQR: interquartile range

Outcome	EFI <7 (n = 22)	EFI ≥7 (n = 26)	p-value
Spontaneous pregnancy, n (%)	2 (9.1)	15 (57.7)	<0.001*
Live birth, n (%)	1 (4.5)	10 (38.5)	-
Time to pregnancy, days, median (IQR)	318.5 (309.8-327.3)	179 (142-214)	-

Factors associated with spontaneous pregnancy

In univariable Cox analysis, increasing age was associated with a lower rate of spontaneous pregnancy (HR per five-year increase 0.42, 95% CI 0.22-0.80; p = 0.008). Higher preoperative AFC was favorable (HR per five follicles 2.30, 95% CI 1.05-5.04; p = 0.036), whereas higher preoperative AMH showed a borderline association (HR per 1 ng/mL 2.32, 95% CI 0.97-5.56; p = 0.060). Ethanol sclerotherapy was associated with a shorter time to pregnancy (HR 4.33, 95% CI 1.66-11.35; p = 0.003). EFI was strongly associated with pregnancy: each one-point increase was associated with an HR of 3.10 (95% CI 1.89-5.08; p < 0.001), and EFI ≥7 was associated with an HR of 9.74 (95% CI 2.21-42.82; p = 0.003). Previous appendectomy was also associated with pregnancy in univariable analysis (HR 2.71, 95% CI 1.04-7.06; p = 0.040), but it was not retained in the primary models because of the absence of a clear biological rationale. Univariable estimates are summarized in Table 5.

**Table 5:** Cox regression for time to spontaneous pregnancy * Statistically significant (p < 0.05). Adjusted estimates shown are from the sensitivity model adjusted for age, AFC, and treatment technique; EFI was not included to avoid redundancy with its historical components. HR: hazard ratio, CI: confidence interval, AMH: anti-Müllerian hormone, AFC: antral follicle count, EFI: Endometriosis Fertility Index

Factor	Unadjusted HR	95% CI	p	Adjusted HR	95% CI	p-value
Age, per 5-year increase	0.42	0.22-0.80	0.008*	0.55	0.27-1.14	0.106
Body mass index, per 5 kg/m²	2.31	0.94-5.66	0.066	-	-	-
Infertility duration, per year	0.92	0.71-1.18	0.510	-	-	-
Secondary vs primary infertility	0.83	0.31-2.24	0.712	-	-	-
Previous ovarian cystectomy	0.88	0.32-2.37	0.797	-	-	-
Previous appendectomy	2.71	1.04-7.06	0.040*	-	-	-
Bilateral endometrioma	0.86	0.33-2.26	0.761	-	-	-
rASRM stage IV vs III	0.87	0.33-2.29	0.780	-	-	-
Preoperative AMH, per 1 ng/mL	2.32	0.97-5.56	0.060	-	-	-
Preoperative AFC, per 5 follicles	2.30	1.05-5.04	0.036*	1.23	0.45-3.40	0.684
Total endometrioma volume, per 20 mL	0.91	0.74-1.12	0.367	-	-	-
Gravidity, per previous pregnancy	0.94	0.57-1.55	0.813	-	-	-
Parity, per previous birth	0.86	0.36-2.03	0.723	-	-	-
Ethanol sclerotherapy vs stripping	4.33	1.66-11.35	0.003*	3.01	1.09-8.28	0.033*
EFI, per 1-point increase	3.10	1.89-5.08	<0.001*	-	-	-
EFI ≥7 vs <7	9.74	2.21-42.82	0.003*	-	-	-

In the primary adjusted model including favorable EFI and treatment technique, EFI ≥7 remained independently associated with spontaneous pregnancy (adjusted hazard ratio (aHR) 7.96, 95% CI 1.78-35.57; p = 0.007), as did ethanol sclerotherapy (aHR 3.11, 95% CI 1.17-8.29; p = 0.023). In an additional model treating EFI as a continuous variable, each one-point increase remained associated with pregnancy (aHR 3.31, 95% CI 1.91-5.73; p < 0.001), and ethanol sclerotherapy remained significant (aHR 4.02, 95% CI 1.48-10.93; p = 0.006). The primary adjusted model is summarized in Table 6 and Figure 1D.

**Table 6:** Primary adjusted Cox model including EFI and treatment technique The small number of pregnancy events justified limiting the model to two predictors. * Statistically significant (p < 0.05). CI: confidence interval, EFI: Endometriosis Fertility Index, HR: hazard ratio

Factor	Adjusted HR	95% CI	p-value
EFI ≥7 vs <7	7.96	1.78-35.57	0.007*
Ethanol sclerotherapy vs stripping	3.11	1.17-8.29	0.023*

In the sensitivity model including age, preoperative AFC, and treatment technique, ethanol sclerotherapy remained associated with pregnancy (aHR 3.01, 95% CI 1.09-8.28; p = 0.033), whereas age (aHR 0.55; p = 0.106) and AFC (aHR 1.23; p = 0.684) were no longer statistically significant. Schoenfeld residual assessment did not suggest a major violation of the proportional hazards assumption for favorable EFI but indicated a time-varying effect of treatment technique; therefore, HRs for sclerotherapy should be interpreted as average effects over follow-up.

## Discussion

In this cohort of 48 infertile women with rASRM stage III-IV endometriosis and histologically confirmed ovarian endometrioma, 17/48 women (35.4%) achieved spontaneous pregnancy after fertility-sparing laparoscopic treatment, and 11/48 (22.9%) achieved a live birth. The median time to conception among women who became pregnant was about six months. Two principal findings emerged. First, a favorable EFI was strongly associated with a higher probability of pregnancy and a shorter time to conception. Second, ethanol sclerotherapy was associated with better reproductive outcomes and more favorable changes in ovarian reserve markers.

The overall spontaneous pregnancy rate is consistent with contemporary literature on fertility after surgery for moderate-to-severe endometriosis. A 2025 cohort of women with rASRM stage III-IV endometriosis reported a spontaneous conception rate of 37.3% after fertility-enhancing minimally invasive surgery [18]. A 2025 systematic review and meta-analysis of nonmedical treatments for endometrioma reported natural pregnancy rates of approximately 40% after cystectomy and 34% after ablative approaches, while emphasizing substantial between-study heterogeneity [16]. The 35.4% rate observed in our cohort therefore falls within the range reported in contemporary studies.

EFI and prognosis of spontaneous pregnancy

The most notable finding of this analysis was the association between EFI and spontaneous pregnancy in 15/26 women (57.7%), compared with 2/22 (9.1%) for EFI <7, and the Kaplan-Meier curves showed marked separation. After adjustment for treatment technique, a favorable EFI remained associated with an approximately eightfold higher rate of spontaneous pregnancy. These findings align with the clinical rationale for the EFI, which combines age, infertility duration, previous pregnancy, and, importantly, residual adnexal function after surgery [3].

The EFI has undergone several external validations. Tomassetti et al. demonstrated a significant relationship between EFI and time to non-ART pregnancy, with cumulative pregnancy probabilities increasing progressively with the score [19]. Among women undergoing surgery for moderate-to-severe endometriosis, Maheux-Lacroix et al. also showed that the EFI predicted live birth, with postoperative adnexal function among the most important determinants [20]. In our cohort, the large HR associated with EFI ≥7, along with its wide CI, should be interpreted in light of the small number of pregnancy events and the limited sample size.

The EFI may be particularly useful in settings where access to assisted reproductive technology is limited. A high score may support a reasonable period of expectant management for spontaneous conception, whereas a low score may justify earlier reassessment and discussion of assisted reproduction. The EFI should nevertheless be calculated from sufficiently detailed operative documentation, particularly for the least function score, rather than reconstructed retrospectively from incomplete information [3,19,20].

Age, ovarian reserve, and probability of spontaneous conception

In univariable analysis, age was associated with pregnancy, with an approximately 58% reduction in the rate of spontaneous pregnancy for each five-year increase. This relationship is biologically plausible, and age is a historical component of the EFI [3]. However, in the sensitivity model adjusted for AFC and treatment technique, the association with age was no longer statistically significant, which may reflect the small sample size, the correlation between age and ovarian reserve, and treatment selection. Gupta et al. also identified age and AFC as factors associated with spontaneous conception after surgery for moderate-to-severe endometriosis [18].

Preoperative AFC was associated with pregnancy in univariable analysis, whereas AMH showed a favorable trend. Zhou et al. reported the predictive value of preoperative AMH for spontaneous pregnancy after endometriosis surgery [21]. In our sensitivity model, AFC lost statistical significance after adjustment, likely reflecting relationships among age, treatment selection, EFI, and ovarian-reserve markers.

Ovarian reserve after stripping

The potential cost of stripping to ovarian reserve is well documented. Randomized and observational evidence suggests that cystectomy is more effective than drainage and coagulation in reducing recurrence, but it may remove normal ovarian cortex and reduce follicular reserve [4-9]. Raffi et al. demonstrated a significant decline in AMH after endometrioma excision [5], Somigliana et al. highlighted a particularly important effect in bilateral disease [6], and Biacchiardi et al. showed a reduction in ovarian reserve even when stripping was performed by experienced surgeons [7]. More recent meta-analyses generally confirm a greater impact of cystectomy on ovarian-reserve markers than less destructive approaches [8-10].

In our stripping group, median AMH did not improve significantly at six months, whereas AFC increased modestly. An increase in AFC after surgery should not be interpreted as new follicle generation; it may reflect improved postoperative visualization, ovarian decompression, intercycle variability, or recovery from perioperative changes. More importantly, changes in both AMH and AFC were substantially greater after sclerotherapy than after stripping.

Ethanol sclerotherapy, ovarian reserve, and spontaneous pregnancy

Sclerotherapy, most commonly performed using ethanol, aims to reduce endometrioma burden while preserving ovarian tissue. Cohen et al. concluded that sclerotherapy may be considered in women wishing to conceive, with clinical pregnancy outcomes comparable with cystectomy in the available studies and a potential advantage in oocyte yield [11]. Martinez-Garcia et al. observed better AFC recovery and spontaneous pregnancies after ethanol sclerotherapy in a prospective cohort [12]. Catheter-directed ethanol sclerotherapy has also been associated with preservation of AMH in women at risk of reduced ovarian reserve [13].

More recent evidence reinforces this signal. Ronsini et al. reported favorable fertility outcomes in a 2023 meta-analysis [14]. Lavadia et al. showed that ethanol sclerotherapy was associated with less AMH impairment than cystectomy [15]. Bennet et al. reported higher postoperative AMH levels after ethanol sclerotherapy than after cystectomy, while emphasizing the high risk of bias and the absence of definitive evidence for a live-birth benefit [22].

In our analysis, 9/15 women (60.0%) in the ethanol sclerotherapy group conceived compared with 8/33 (24.2%) after stripping, and pregnancies occurred earlier. Sclerotherapy remained associated with pregnancy in the model that included EFI. However, the treatment groups were not comparable at baseline: women selected for sclerotherapy were younger, had higher AFC, and more frequently had favorable EFI scores. Even after adjustment, selection bias and residual confounding remain likely. Additionally, the effect of treatment technique varied over time. The HR for sclerotherapy should therefore not be interpreted as a constant causal effect.

Clinical implications

These data support an individualized fertility-preserving strategy. Stripping remains an established option when complete cyst-wall removal, reduction of recurrence risk, histologic assessment, or symptom control is prioritized [2,4]. In women with diminished ovarian reserve, bilateral disease, previous ovarian surgery, or a strong desire to conceive in the near term, a less destructive approach may be considered. The EFI adds a prognostic dimension by integrating preoperative characteristics and residual adnexal function to help guide the duration of expectant management before referral for assisted reproductive technology [3,19,20].

All spontaneous pregnancies in this cohort occurred within 336 days. For women who have not conceived within one year after treatment, particularly those with a low EFI, advanced reproductive age, or diminished ovarian reserve, early reassessment and discussion of assisted reproductive technology may be appropriate, consistent with individualized guideline-based care [2,3].

Strengths and limitations

Strengths include histopathologic confirmation in all women, measurement of AMH and AFC before treatment and at six months, direct comparison of two fertility-sparing strategies, incorporation of the EFI, and time-to-pregnancy analysis. Because the same surgical team performed all procedures, operator-related variability was reduced.

Several limitations should be acknowledged. First, the retrospective, nonrandomized design introduces selection bias and limits causal inference. Second, the treatment-technique effect did not fully satisfy the proportional hazards assumption, indicating that its association with pregnancy varied over time. Third, the small sample size and limited number of pregnancy events reduce statistical precision and generalizability. Larger prospective, multicenter studies are required to confirm these findings.

## Conclusions

In this cohort of 48 infertile women with advanced endometriosis and ovarian endometrioma, approximately one-third achieved spontaneous pregnancy after laparoscopic treatment, predominantly within the first year. A higher EFI was strongly associated with a more favorable reproductive prognosis and remained significant after adjustment for treatment technique. Ethanol sclerotherapy was also associated with more frequent spontaneous pregnancy and more favorable ovarian-reserve markers than stripping.

These findings support the integration of ovarian-preserving treatment strategies with postoperative prognostic assessment using the EFI. The EFI may help identify women for whom a period of expectant management for spontaneous conception is reasonable and those who may benefit from earlier referral for assisted reproductive technology. The association observed with ethanol sclerotherapy should, however, be interpreted as an association rather than proof of a causal treatment effect because treatment allocation was not randomized.
